# Novel Neutrophilic Parameters of the Sysmex XN-1000V for the Prediction of Inflammation in Dogs

**DOI:** 10.3390/ani15223275

**Published:** 2025-11-12

**Authors:** Leandra C. Schöb, Melanie Ginder, Martina Stirn, Regina Hofmann-Lehmann, Heiner M. Hipp, Barbara Riond

**Affiliations:** 1 Clinical Laboratory, Department for Clinical Services and Diagnostics, Vetsuisse Faculty, University of Zurich, 8057 Zurich, Switzerland; leandrachristina.schoeb@uzh.ch (L.C.S.); rhofmann@vetclinics.uzh.ch (R.H.-L.); 2 Sysmex Europe SE, 22297 Norderstedt, Germany; melanie.ginder@sysmex-europe.com; 3 Roche Pharma Research and Early Development, Roche Innovation Center, 4070 Basel, Switzerland; martina.stirn@roche.com; 4 Clinic for Small Animal Internal Medicine, Vetsuisse Faculty, University of Zurich, 8057 Zurich, Switzerland; heiner.hipp@uzh.ch

**Keywords:** canine, diagnostics, hematology, immune response, inflammation, marker, neutrophilic parameters, SIRS, Sysmex XN-1000V

## Abstract

Background: Inflammation is a common reason for dogs to present to veterinary clinics. Early diagnosis of systemic inflammation is important. Acute phase proteins, like C-reactive protein, are useful but not specific to infection. In human medicine, the intensive care infection score (ICIS) offers a faster, cost-effective alternative using advanced hematological parameters. While ICIS is not available for veterinary use, some components (e.g., neutrophil side fluorescent light) can be measured using analyzers like the Sysmex XN-1000V. Objectives: This study aimed to establish a control group of healthy dogs for the novel parameters neutrophil side fluorescent light (NE-SFL), neutrophil side scattered light (NE-SSC), and neutrophil forward scattered light (NE-FSC) and assess their utility in detecting inflammation in diseases such as sepsis, pyometra, steroid-responsive meningitis-arteritis (SRMA), and idiopathic epilepsy. Methods & Results: Value ranges were calculated based on 21 healthy dogs. Compared to controls, NE-SFL levels were significantly elevated in sepsis, pyometra, and SRMA, while NE-SSC was only elevated in sepsis and pyometra and NE-FSC only in sepsis. No increases were observed in idiopathic epilepsy. Manual gating of the white blood cell differential scattergram was necessary in samples showing high neutrophil toxicity and the presence of bands. Conclusion: NE-SFL and NE-SSC, obtainable from routine complete blood count, may serve as novel, accessible markers for inflammation in dogs. Further research is needed to validate their broader diagnostic use.

## Simple Summary

In veterinary medicine, dogs often suffer from various inflammatory and non-inflammatory diseases. For effective case management, rapid diagnosis of any underlying inflammation is essential. Routine diagnostics include testing of different blood parameters, such as C-reactive protein or complete blood count. Novel neutrophilic parameters, like neutrophil side fluorescent light (NE-SFL), neutrophil side scattered light (NE-SSC) and neutrophil forward scattered light (NE-FSC) measured on the Sysmex XN-1000V are promising values for detection of inflammation in dogs. In the first part of this study, a control group of healthy dogs was established for the novel neutrophilic parameters. In the second part, the performance of these novel parameters was evaluated in inflammatory and non-inflammatory diseases. The study showed that these parameters can effectively differentiate between inflammatory and non-inflammatory conditions, with NE-SFL being the most consistent marker. It was strongly correlated with the severity of inflammation. However, manual corrections were sometimes needed for accurate analysis, especially in severe cases, which require trained staff with appropriate expertise. In conclusion, NE-SFL and NE-SSC demonstrated promising performance as novel, cost-effective markers of inflammation in dogs and may improve diagnosis of inflammation in veterinary practice.

## 1. Introduction

Cell injury or death caused by infectious and non-infectious agents, physical trauma, extreme temperatures (heat or cold), radiation, or malignant cells can trigger a highly coordinated sequence of fluid and cellular responses within vascularized tissues, a process known as inflammation. This response leads to the buildup of fluid, electrolytes, plasma proteins, and leukocytes in extravascular tissue. Clinically, inflammation is identified by redness, heat, swelling, pain, and loss of function in the affected tissue. It typically acts as a defense mechanism, aiming to dilute, contain, and remove the cause of injury, while also initiating tissue repair. Leukocytes play an essential role in this process, forming a key part of the immune system and defending the body against harmful pathogens, including bacteria, viruses, fungi, and parasites [1].

In human medicine, the intensive care infection score (ICIS) has been investigated for critically ill patients to differentiate between infectious and non-infectious processes [2]. ICIS comprises five blood-cell-derived parameters: the mean fluorescence intensity of mature (segmented) neutrophils (NE-SFL), the difference in hemoglobin concentration between new (RET-H_e_) and mature (RBC-H_e_) red blood cells (Delta-H_e_), total segmented neutrophil count, antibody-secreting lymphocytes, and immature granulocyte count [3]. In clinical settings, physicians may encounter challenges in promptly establishing a diagnosis, often due to inconclusive clinical findings, insufficient diagnostic markers, or delays in obtaining microbiological results [2,4]. Measurement of acute-phase markers, like C-reactive protein (CRP), but especially procalcitonin (PCT), are time-consuming and costly [5]. There is a need for new and reliable approaches for the rapid diagnosis of sepsis [2]. Studies in humans have shown that the ICIS is potentially useful for the prediction of infection and its severity in critically ill patients [3], and that it serves as a reliable marker with comparable or higher sensitivity and specificity than CRP and PCT [2]. Furthermore, ICIS offers several advantages over conventional inflammatory markers such as CRP or PCT. One benefit is that no additional blood sample is needed, provided that a white blood cell (WBC) analysis has already been performed using a K_3_EDTA sample. In this case, ICIS can be measured from the same tube. Another advantage lies in the low associated costs, as ICIS is measured on the same analyzer used for WBC assessment [3].

In veterinary medicine, dogs are often presented to veterinary clinics due to a wide range of health issues. Inflammation is often the underlying cause of the condition. Canine WBC counts and their specific subpopulations can vary significantly during inflammation, primarily due to interactions between the immune system and the pathogen, as well as the host’s inflammatory response. Therefore, information about the leukogram, including WBC count, leukocyte differential, and morphological changes in leukocytes, provides valuable insights into the type and stage of inflammation in dogs, and has been used for decades to diagnose inflammation in the canine patient. The leukogram is moderately sensitive in detecting inflammation; therefore, a normal leukogram does not rule out inflammatory disease [6]. Measurement of acute phase proteins, such as CRP in dogs, is a more sensitive test. CRP increases rapidly as part of a number of inflammatory diseases [7,8] and plays important roles by protecting against infection, clearance of damaged tissue, prevention of autoimmunization, and regulation of the inflammatory response [9].

In contrast to human medicine, the evaluation of morphological WBC changes in dogs relies on the examination of a stained blood smear, which is time-consuming and depending on additional laboratory equipment (such as staining capabilities, a microscope) and trained staff. A more rapidly responding parameter for predicting inflammation in dogs, that could be analyzed faster and cheaper, would therefore be beneficial.

ICIS is currently not available for animal patients. However, individual parameters, such as mean fluorescence intensity of neutrophils (NE-SFL) and difference in hemoglobin concentration between immature and mature red blood cells (Delta-H_e_), are available. Modern hematological analyzers, such as the Sysmex XN-V Series, are capable of measuring more parameters than the traditional complete blood cell count [10]. In the white blood cell differential (WDF) channel of the Sysmex XN-1000V hematology device (Sysmex Corporation, Kobe, Japan), fluorescence flow cytometry is applied to measure neutrophilic granulocytes among other white blood cells. In this channel, cells are differentiated based on side fluorescent light (SFL), side scattered light (SSC), and forward scattered light (FSC) signals [11]. Depending on individual cell characteristics, differences in these light and fluorescence signals might be detected and reported as NE-FSC, NE-SSC, and NE-SFL values. Information about the performance of these novel parameters in inflammatory or infectious and non-inflammatory diseases in dogs, and whether or not they can be an early and effective marker of inflammation, is lacking. A first study by O’Toole et al. suggests that NE-SSC, and particularly NE-SFL, may serve as promising markers of systemic inflammation in dogs and cats [12].

The aim of this study was to evaluate retrospectively three novel neutrophilic granulocyte parameters NE-SFL, NE-SSC, and NE-FSC in healthy dogs and dogs with and without inflammatory diseases, and to compare these parameters with established inflammatory markers such as CRP, WBC count, the presence of bands, and toxic changes.

For this, a control group of healthy dogs was determined (1) and different disease groups were established (septic dogs, dogs with pyometra, steroid-responsive meningitis-arteritis (SRMA) and idiopathic epilepsy) to compare NE-SFL, NE-SSC and NE-FSC values with traditional inflammatory markers (CRP concentration, total WBC count, presence of bands and neutrophilic toxic changes) (2).

## 2. Materials and Methods

### 2.1. Novel Neutrophilic Granulocyte Parameters NE-SFL, NE-SSC, and NE-FSC from Sysmex XN-1000V

The Sysmex XN-1000V veterinary hematology analyzer (Sysmex Corporation, Kobe, Japan) is the latest hematological high-throughput device on the veterinary market and is derived from the Sysmex XN Series technological platform. It is the successor of the Sysmex XT-2000iV and is characterized by important new features, including an optic-fluorescent analysis for platelets (platelet fluorescence channel, using an oxazine-based fluorescent dye), the counting of nucleated red blood cells in the WNR-channel [13] with every complete blood count and a body fluid application to measure fluids such as cerebrospinal and bronchoalveolar lavage fluids [14,15,16]. Generally, the WDF channel differentiates leukocytes and therefore counts neutrophils, lymphocytes, monocytes, and eosinophils through fluorescence flow cytometry, utilizing a red semiconductor laser with a wavelength of 633 nm [11]. In this process, leukocytes are first permeabilized using a surfactant and then stained with a fluorescent polymethine dye, which binds to cytoplasmatic organelles and nucleic acids (DNA and RNA) [17]. By doing this, three signals are recorded in the white blood cell differential scattergram: side fluorescent light (SFL, DNA/RNA content), side scattered light (SSC, granularity), and forward scattered light (FSC, cell size) [18]. In the same process, novel parameters, such as the NE-FSC, NE-SSC, and NE-SFL, are determined as research parameters that could be displayed on customizable User Screens within the instrument’s Browser section of the IPU.

NE-SFL is considered an indicator of neutrophil activation, as the fluorescent dye binds to nucleic acids within both cytoplasmic organelles and the nucleus in activated cells [17,19]. Its intensity corresponds to the composition and quantity of nucleic acids and cellular organelles [20].

NE-SSC measures the nucleus and internal granularity of neutrophils [21]. When neutrophil complexity increases due to functional changes, such as toxic granulation or vacuolization, their position in the scattergram shifts accordingly [22].

The NE-FSC signal detects cell size [21]. Activated neutrophils may show an abnormal cell size, leading to an altered FSC signal [23].

The positioning of the neutrophil granulocyte population in the scattergram allows an evaluation of the activity of the neutrophils [22] and generates the parameters NE-SFL, NE-SSC, and NE-FSC.

Prior to the measurement of study samples, internal quality control was performed once daily by measuring control material provided by the manufacturer (XN-Check L1-L3). The controls were processed like patient samples and measured on a properly calibrated and fully functional device in quality control mode. The measurement results must fall within the assay ranges specified on the assay sheet. Control materials were stored at a temperature of 2–8 °C and warmed up to room temperature for analysis.

### 2.2. Blood Smear Evaluation

Blood smear preparation was performed within one hour after sample collection by trained laboratory technicians. The smears were air-dried for 20 min before staining. Staining was performed on a HEMA-TEK 2000 slide stainer (Siemens Healthcare GmbH, Erlangen, Germany) with modified Wright’s stain. Two experienced laboratory technicians performed a 200 WBC differential by microscopic blood smear evaluation, using a Leica DM LB2 microscope (Leica Microsystems AG, Heerbrugg, Switzerland) with 50× and 100× oil immersion objectives. Relative and absolute numbers of bands were determined. Bands were defined with unsegmented and smooth nuclear walls and immature chromatin structure. The laboratory’s reference interval ranged from 0 to 0.084 × 10^3^/µL.

Additionally, the quantity and degree of toxic changes in neutrophils were assessed semiquantitatively. The presence of Döhle bodies, foamy cytoplasm, basophilic cytoplasm and toxic granulation indicated toxicity. The quantity and the degree were judged according to the grading scheme of Harvey [24]. Toxic changes in neutrophils were not observed in the healthy control group.

### 2.3. Measurement of Canine C-Reactive Protein (CRP)

CRP concentrations were determined on a fully automated chemistry analyzer (Cobas C 501, Roche Diagnostics, Rotkreuz, Switzerland) using two commercially available test kits validated for the canine species (Randox Laboratories Ltd., Crumlin, UK and Gentian Canine CRP Immunoassay, Orlando, USA), which have been previously validated [25,26]. The cut-off value for healthy control dogs was ≤10.7 mg/L (Randox Laboratories Ltd., UK).

The study population was retrospectively assembled from cases between 2018 and 2024. Until December 2022, CRP concentrations were measured using the Randox assay; from January 2023 onward, the Gentian assay was used. The cut-off values applied were >10.7 mg/L for Randox and >10.2 mg/L for Gentian.

### 2.4. Establishment of a Control Group for NE-SFL, NE-SSC, and NE-FSC

In the first part of this study, 21 healthy dogs were prospectively selected to establish a control group for the novel neutrophilic granulocyte parameters. The following criteria had to be met: the dogs had to be anamnestically and clinically healthy, free of medication for at least one month, with CRP concentration, WBC count, and neutrophil cell count within the reference range and absence of left shift and toxic changes. To ensure this, dog owners were interviewed about their pets’ health status, medication history, and additional details, such as breed, age, gender, and neuter status, were recorded. Dogs that did not meet these criteria or had values outside the reference ranges were excluded from the healthy control group. Neither gender nor neuter status served as exclusion criteria for the healthy dogs; however, they had to be at least one year old to eliminate the influence of age-related blood changes observed in younger dogs [27]. Greyhounds and Greyhound mix breeds were excluded from the study due to their well-documented clinicopathologic differences, particularly in hematologic profiles, which distinguish them from other dog breeds [28].

All healthy dogs of the control group were privately owned, and their owners provided written consent for study participation. Approval from the Cantonal Veterinary Office (ZH 057/19, Zurich, Switzerland) was obtained prior to conducting the study. Before blood sampling, each dog underwent a brief physical examination. Venous blood was then collected using a 20-gauge (0.9 × 25 mm) needle from either the cephalic vein (*Vena cephalica antebrachii*) of the front leg or the saphenous vein (*V. saphena*) of the hind leg, without sedation. If necessary, the puncture site was shaved and disinfected beforehand. Blood was drawn into sterile K_3_EDTA tubes (1.3 mL) and serum tubes (4 mL). After 30 min, whole blood samples were centrifuged at 2000× *g* (Rotina 380 R, Hettich, Bäch, Switzerland), and the serum was subsequently collected. All blood samples were analyzed within four hours at the Clinical Laboratory of the Vetsuisse Faculty, University of Zurich, Switzerland. A complete blood count, including WBC differentiation, was performed using the Sysmex XN-1000V hematology analyzer (Sysmex Corporation, Kobe, Japan), and blood smears were evaluated for the presence of platelet clumps and morphological abnormalities. CRP concentrations were determined using a canine CRP assay (Randox Laboratories Ltd., UK).

### 2.5. Data Collection for Study Group

In the second part of this study, the performance of the novel neutrophilic granulocyte parameters in inflammatory and non-inflammatory diseases was retrospectively evaluated in a total of 84 diseased dogs, comprising the study population. The study population was organized into two main groups. Group 1 covered dogs with non-inflammatory diseases and contained 20 dogs with idiopathic epilepsy. Group 2 enclosed dogs with inflammatory diseases, and the corresponding subgroups contained 23 dogs with SRMA, 20 dogs with pyometra, and 21 dogs with sepsis or septic abdomen.

The selection of animals in the study group was performed retrospectively between January 2018 and May 2024, based on the diagnosis of board-certified specialists. Inclusion criteria for all subgroups were as follows: (1) CRP concentration were measured, (2) WBC count and the novel neutrophilic granulocyte parameters (NE-SFL, NE-SSC, and NE-FSC) were measured on the Sysmex XN-V hematology analyzer, and (3) WBC differentiation (including count of bands) and toxic changes (quantity and degree) were evaluated manually on stained blood smears. All parameters were analyzed on the day of admission to the clinic for small animals, Vetsuisse Faculty, University of Zurich, Switzerland. Dogs were excluded if any data was lacking.

The diagnosis of idiopathic epilepsy was based on the anamnesis (and ruling out other possible causes of seizures), neurological exam, and further diagnostics, like analysis of the cerebrospinal fluid or results from MRI/CT. The diagnosis of SRMA was based on clinical signs (neck stiffness, neck pain and fever without neurological deficits), cerebrospinal fluid analysis and a positive response to corticosteroid therapy. The diagnosis of pyometra was based on bacteriological culture of uterine contents, with additional support from hematological and biochemical findings, as well as abdominal imaging (ultrasound and/or radiographs). Diagnosis of sepsis was based on the following two criteria: (1) Having two or more systemic inflammatory response syndrome (SIRS) criteria. The SIRS-criteria utilized were: temperature <38.1 °C/>39.2 °C, heart rate >120/min., respiratory rate >20/min., WBC count <6 × 10^3^/µL or >16 × 10^3^/µL or bands >3% [29]. (2) Testing positive for bacterial culture in an abdominal organ (excluding the urogenital tract) or the peritoneal cavity.

Medical records of all subgroups were extracted from the clinical information system (Vetera GmbH, Eltville am Rhein, Germany). Blood analysis results were extracted from the laboratory information systems (Vianova, Clinisys, Gent, Belgium), along with hematology data from the Sysmex XN-1000V instrument of the Clinical Laboratory of the Vetsuisse Faculty of Zurich, Switzerland.

### 2.6. Manual Gating

When the notification “WBC Abn Scattergram” is given by the Sysmex XN-V with the result report, it points towards an abnormal pattern of the cell clusters displayed in the scattergram. General reasons could be alterations in cell morphology, presence of immature, reactive, atypical, or neoplastic cells, as well as the occurrence of interfering conditions (e.g., marked platelet clumping). In our case, most abnormal scattergram flags occurred due to the presence of immature neutrophilic granulocytes in the sample or because the neutrophils showed cytoplasmic toxicity. In some cases, these neutrophils were misclassified as monocytes or monocytes and lymphocytes. For these cases, manual gating was performed using the instrument’s Manual Analysis software tool (XN-1000V, software version 3.07). Applying the new gates corrected the cell count, as well as the respective neutrophil values NE-SFL, NE-SSC, and NE-FSC. The newly created gates were saved as a new analysis profile to reapply them to similar cases. This approach eliminated the need to perform gating from scratch for each sample; instead, only a cross-check was required to ensure that the cell clusters were properly positioned within the applied gates. If not, the respective gates could be adjusted as needed.

### 2.7. Statistical Analysis

The statistics of the control group were generated using the Analyse-it (version 6.15, Analyse-it Software Ltd., Leeds, UK), an add-in for Microsoft Excel (version 2021, Microsoft Corporation, Redmond, WA, USA). For the control group, minimum, maximum, and median values were calculated for Ne-SFL, NE-SSC, and NE-FSC.

Statistical analysis for the study groups and comparisons between the study groups and the healthy control group were performed using GraphPad Prism 10 (version 10, GraphPad Software, San Diego, CA, USA). To test small sample sizes (<50) for normal distributions, the Shapiro–Wilk test is recommended [30] and was used. It demonstrated that the data of all groups were mostly not normally distributed; therefore, nonparametric tests were used [31]. Group comparisons between all five groups were performed using the Kruskal–Wallis test (p_KW_), followed by Dunn’s post hoc test (p_Dunn_) [31]. Spearman’s correlation coefficient (p_S_) was used [32] to assess the correlations between NE-SFL, NE-SSC, NE-FSC, and other hematological parameters, namely the WBC count, CRP concentration, count of bands, and toxic changes. The strength of the correlation was interpreted as follows: coefficients between 0.0 and 0.1 were considered to indicate no correlation, 0.1 to 0.3 a low correlation, 0.3 to 0.5 a moderate correlation, 0.5 to 0.7 a strong correlation, and values above 0.7 a very strong correlation [33]. For all calculated *p*-values (p_KW_, p_Dunn_, p_S_,), a significance threshold of *p* < 0.05 was applied.

Descriptive statistics for the study groups (median and interquartile range) were calculated using Microsoft Excel (version 2021, Microsoft Corporation, Redmond, WA, USA) with built-in formulas.

## 3. Results

### 3.1. Control Group

The 21 healthy dogs of the control group were between one and eleven years of age. This group included one Eurasier, one Retro Pug, one Standard Schnauzer, one Border Collie, two Medium Poodles, one Havanese, one Nova Scotia Duck Tolling Retriever, one Border Terrier, one Shetland Sheepdog, one German Spitz, one Cocker Spaniel, one Flat Coated Retriever, one Boxer and seven mixed breeds. Of the 21 dogs, eleven were female and ten were male. Three dogs were not castrated (one female and two male dogs), and two of the 18 castrated dogs were chemically neutered.

For establishing the neutrophilic granulocyte parameters of the healthy dogs, no manual gating was needed (Figure 7A). The minimum, maximum, and median values are displayed in Table 1. Value ranges for NE-SFL, NE-SSC, and NE-FSC were 38.2–42.7 channel value (ch), 92.6–106.6 ch, and 38.7–53.4 ch, respectively.

### 3.2. Performance of NE-SFL, NE-SSC, and NE-FSC in Different Disease Categories

In the SRMA and idiopathic epilepsy study groups, manual gating was not required (Figure 7B,C). However, in the pyometra group, twelve out of 20, and in the sepsis group, eight out of 21, required manual gating (Figure 7D–I).

Table 2 presents the median values and interquartile ranges for CRP concentration, total WBC count, count of bands, NE-SFL, NE-SSC, and NE-FSC across each disease category and the control group, along with the corresponding reference intervals. Table 3 provides an overview of the occurrence of toxic changes in every disease category and the control group.

**Figure 1:**
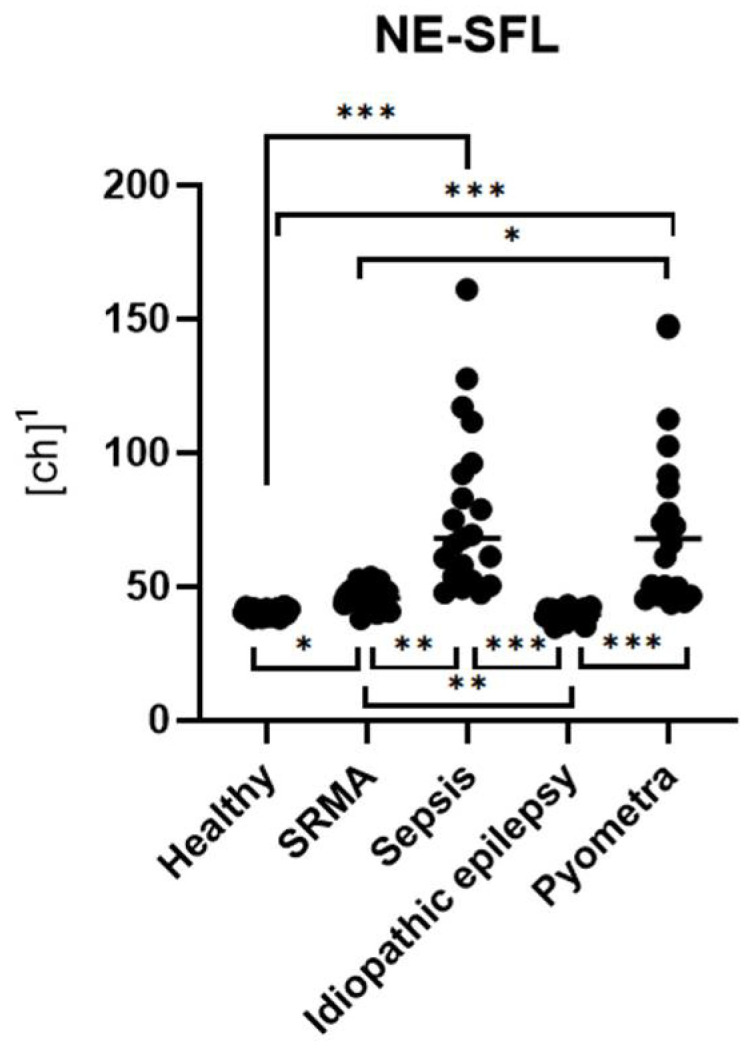
Group comparisons of NE-SFL levels across different disease categories with corresponding statistically significant differences (p_Dunn_). Data from each group are presented as single dots; the vertical line represents the median. The groups were compared using the Kruskal–Wallis test, followed by Dunn’s post hoc test. ^1^ ch = channel value (technical value, describing position in the axis).

There was a significant difference in NE-SFL among the five groups (p_KW_ < 0.001, ***) (Figure 1). NE-SFL was significantly higher in dogs with sepsis and pyometra (p_Dunn_ < 0.001, ***), as well as in dogs with SRMA (p_Dunn_ = 0.039, *), compared to the healthy control group (Figure 1). Dogs with idiopathic epilepsy did not show a significant difference in NE-SFL compared to the healthy control group (p_Dunn_ > 0.999, ns). Additionally, NE-SFL levels were significantly higher in dogs with sepsis or pyometra than in those with idiopathic epilepsy (p_Dunn_ < 0.001, ***) or with SRMA (p_Dunn_ = 0.005, **; p_Dunn_ = 0.043, *, respectively) (Figure 1). However, there was no significant difference between dogs with sepsis and those with pyometra (p_Dunn_ > 0.999, ns). Furthermore, NE-SFL levels were significantly higher in dogs with SRMA than in those with idiopathic epilepsy (p_Dunn_ = 0.003, **) (Figure 1).

**Figure 2:**
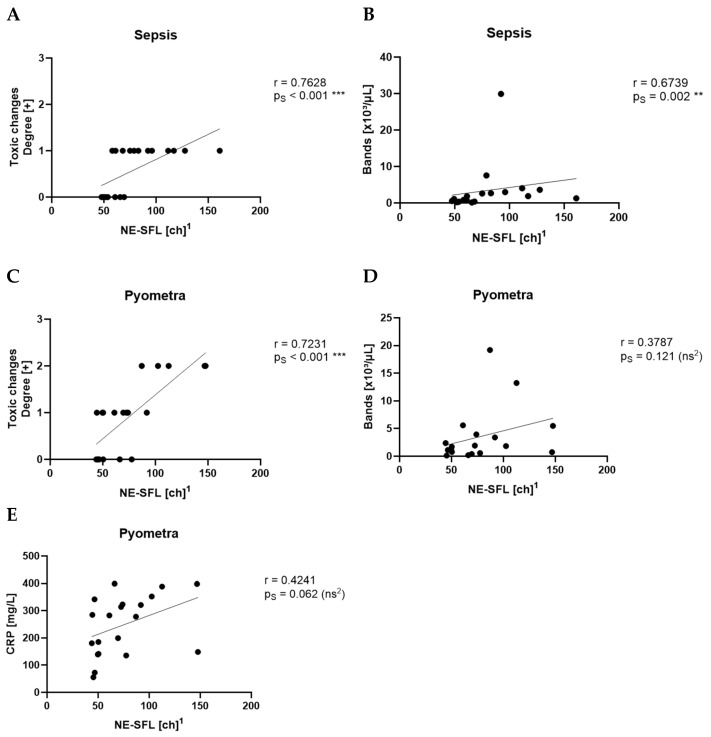
Spearman’s correlation (p_S_) of NE-SFL with the degree of toxic changes (**A**) and with the count of bands (**B**) in the sepsis group. Spearman’s correlation of NE-SFL with the degree of toxic changes (**C**), the count of bands (**D**), and CRP levels (**E**) in the pyometra group. ^1^ ch = channel value (technical value, describing position in the axis). ^2^ ns = not statistically significant.

NE-SFL showed a very strong correlation with toxic changes (r = 0.7628, p_S_ < 0.001, ***) and a strong correlation with the count of bands (r = 0.6739, p_S_ = 0.002, **) in the sepsis group (Figure 2A,B). In the pyometra group, NE-SFL correlated very strongly with toxic changes (r = 0.7231, p_S_ < 0.001, ***) (Figure 2C), and showed no significant correlations with the count of bands (r = 0.3787, p_S_ = 0.121, ns) (Figure 2D) and CRP (r = 0.4241, p_S_ = 0.062, ns) (Figure 2E). Beyond these findings, no other parameters demonstrated a moderate or strong correlation with NE-SFL in the sepsis and pyometra groups. There was no moderate or strong correlation between NE-SFL and any other parameter in the SRMA or idiopathic epilepsy groups.

**Figure 3:**
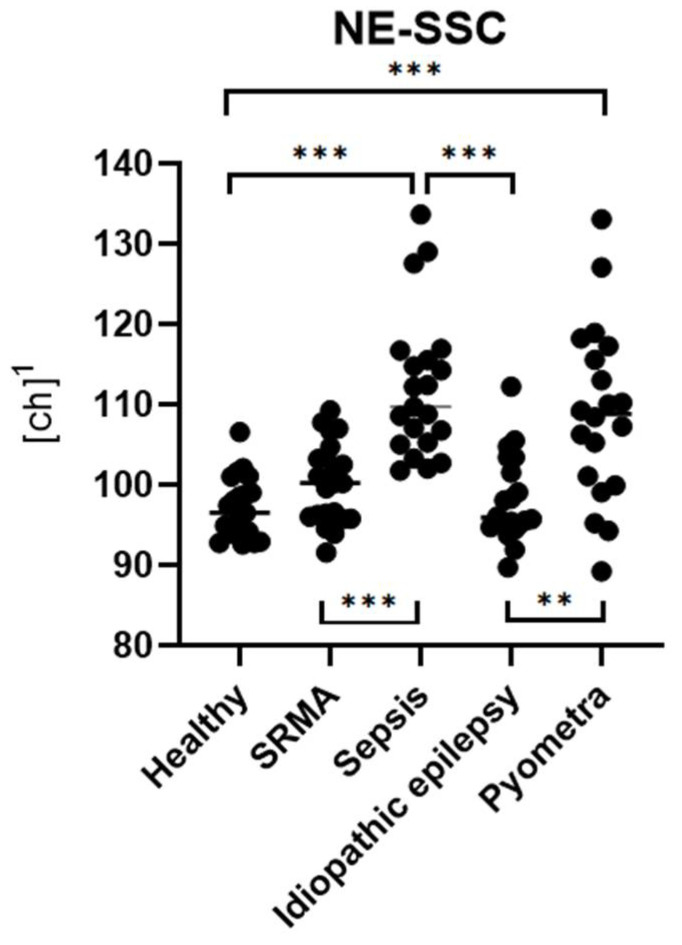
Group comparisons of NE-SSC levels across different disease categories with corresponding statistically significant differences (p_Dunn_). Data from each group are presented as single dots; the vertical line represents the median. The groups were compared using the Kruskal–Wallis test, followed by Dunn’s post hoc test. ^1^ ch = channel value (technical value, describing position in the axis).

There was a significant difference in NE-SSC among the five groups (p_KW_ < 0.001, ***) (Figure 3). NE-SSC levels were significantly higher in dogs with sepsis and pyometra compared to the healthy control group (p_Dunn_ < 0.001, ***) (Figure 3). However, NE-SSC levels were not significantly higher in dogs with SRMA or idiopathic epilepsy compared to the healthy control group (p_Dunn_ > 0.999, ns). NE-SSC levels showed significant differences in the sepsis group compared to the idiopathic epilepsy and SRMA groups (p_Dunn_ < 0.001, ***) (Figure 3). In the pyometra group, Ne-SSC levels were only significantly higher compared to the idiopathic epilepsy group (p_Dunn_ = 0.002, **) (Figure 3), but not to the SRMA group (p_Dunn_ = 0.068, ns). There were no significant differences in the NE-SSC levels between the sepsis and pyometra groups and between the SRMA and idiopathic epilepsy groups (p_Dunn_ > 0.999, ns).

**Figure 4:**
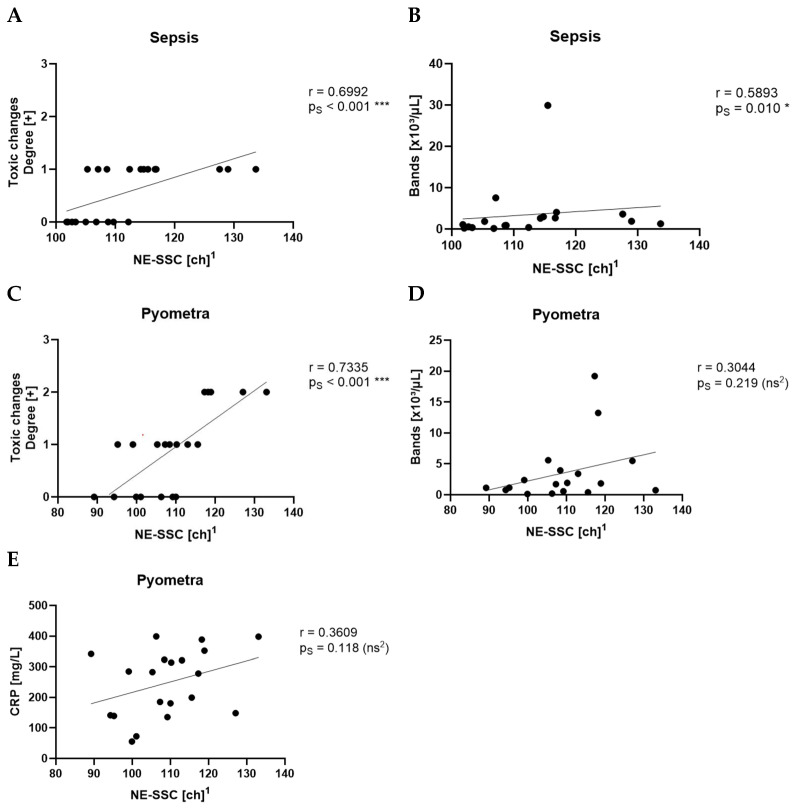
Spearman’s correlation (p_S_) of NE-SSC with the degree of toxic changes (**A**) and with the count of bands (**B**) in the sepsis group. Spearman’s correlation of NE-SSC with the degree of toxic changes (**C**), the count of bands (**D**), and CRP levels (**E**) in the pyometra group. ^1^ ch = channel value (technical value, describing position in the axis). ^2^ ns = not statistically significant.

NE-SSC showed a very strong correlation with toxic changes in the pyometra group (r = 0.7335, p_S_ < 0.001, ***) (Figure 4C) and a strong correlation with toxic changes in the sepsis group (r = 0.6992, p_S_ < 0.001, ***) (Figure 4A). In the sepsis group, NE-SSC also strongly correlated with the count of bands (r = 0.5893, p_S_ = 0.010, *) (Figure 4B). Additionally, in the pyometra group, Ne-SSC showed no significant correlation with the count of bands (r = 0.3044, p_S_ = 0.219, ns) (Figure 4D) and with CRP (r = 0.3609, p_S_ = 0.118, ns) (Figure 4E). Beyond these findings, no other parameters demonstrated a moderate or strong correlation with NE-SSC in the sepsis and pyometra groups. There was no moderate or strong correlation between NE-SSC and any other parameter in the SRMA or idiopathic epilepsy groups.

**Figure 5:**
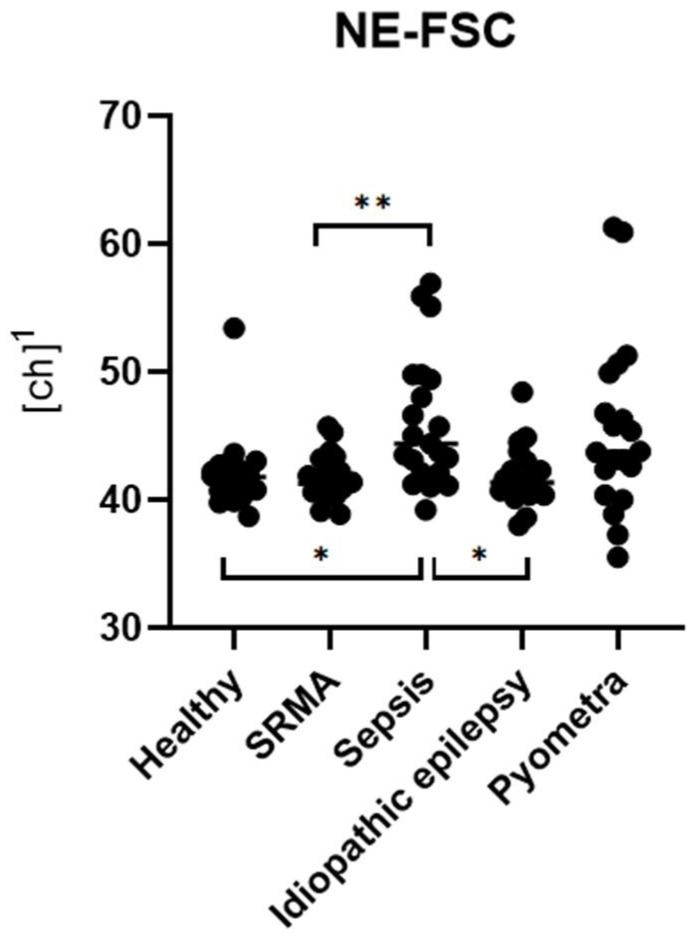
Group comparisons of NE-FSC levels across different disease categories with corresponding statistically significant differences (p_Dunn_). Data from each group are presented as single dots; the vertical line represents the median. The groups were compared using the Kruskal–Wallis test, followed by Dunn’s post hoc test. ^1^ ch = channel value (technical value, describing position in the axis).

There was a significant difference in NE-FSC among the five groups (p_KW_ < 0.001, ***) (Figure 5). In contrast, NE-FSC levels were significantly higher only in dogs with sepsis compared to the healthy control group (p_Dunn_ = 0.015, *) (Figure 5). No significant differences in NE-FSC were observed in dogs with pyometra (p_Dunn_ = 0.187, ns), SRMA (p_Dunn_ > 0.999, ns) or idiopathic epilepsy (p_Dunn_ > 0.999, ns) compared to the healthy control group.

In the sepsis group, NE-FSC levels were significantly higher compared to the SRMA (p_Dunn_ = 0.010, **) and idiopathic epilepsy groups (p_Dunn_ = 0.016, *) (Figure 5) but were not significantly different from the pyometra group (p_Dunn_ > 0.999, ns). There was no significant difference in the NE-FSC levels in the pyometra group compared to either the SRMA group (p_Dunn_ = 0.142, ns) or idiopathic epilepsy group (p_Dunn_ = 0.193, ns). There was no significant difference in the NE-FSC levels between the SRMA and idiopathic epilepsy groups (p_Dunn_ > 0.999, ns).

**Figure 6:**
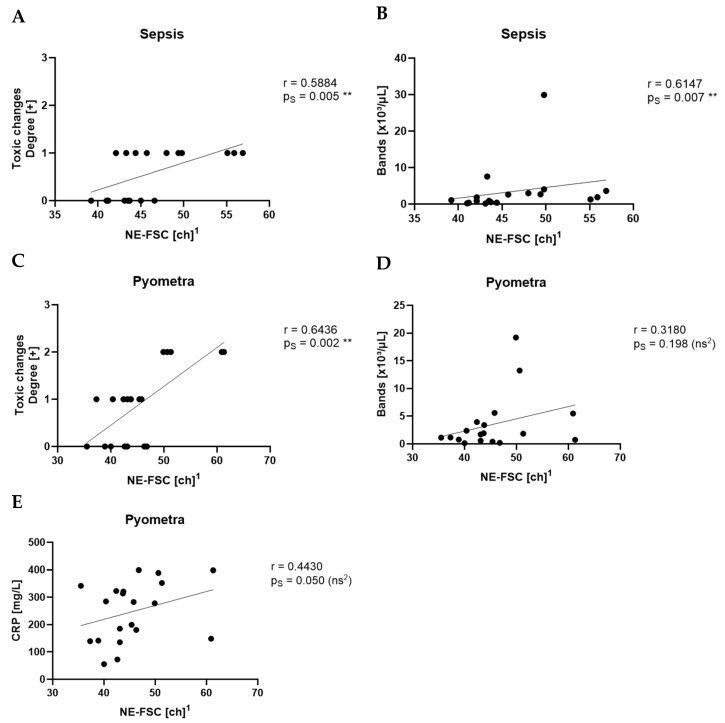
Spearman’s correlation (p_S_) of NE-FSC with the degree of toxic changes (**A**) and with the count of bands (**B**) in the sepsis group. Spearman’s correlation of NE-FSC with the degree of toxic changes (**C**), the count of bands (**D**), and CRP levels (**E**) in the pyometra group. ^1^ ch = channel value (technical value, describing position in the axis) ^2^ ns = not statistically significant.

**Figure 7:**
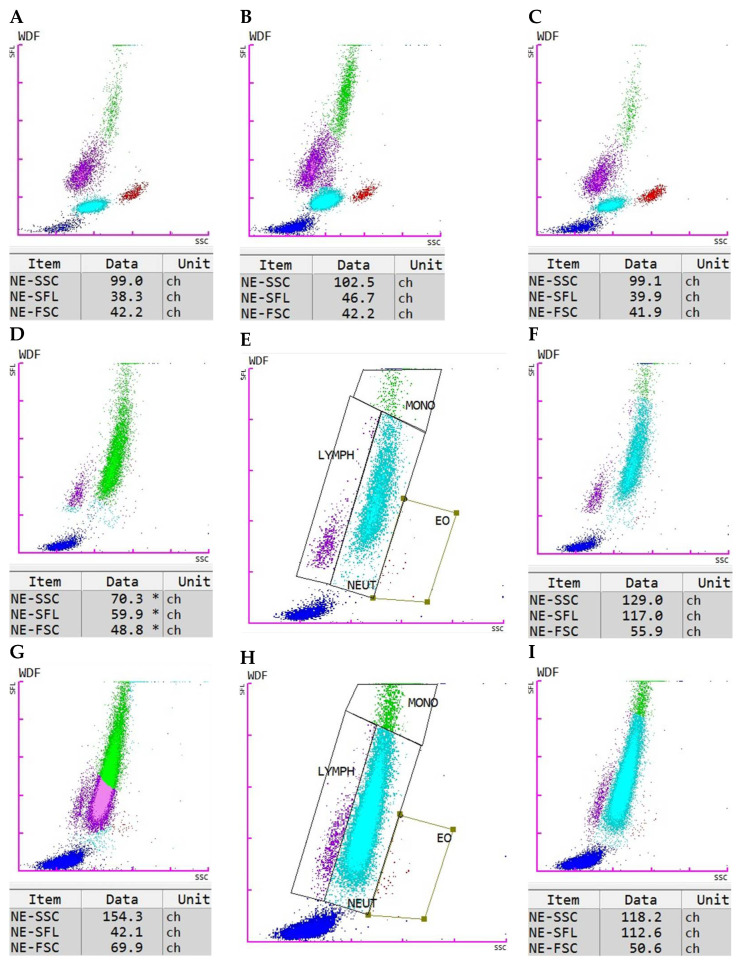
Scattergram of a healthy dog (**A**), a dog with SRMA (**B**), and a dog with idiopathic epilepsy (**C**) with corresponding NE-SFL, NE-SSC, and NE-FSC levels. Scattergram of a dog with sepsis before manual gating (**D**), with applied gates (**E**), and after manual gating with corrected NE-SFL, NE-SSC, and NE-FSC levels (**F**). Scattergram of a dog with pyometra before manual gating (**G**), with applied gates (**H**), and after manual gating with corrected NE-SFL, NE-SSC, and NE-FSC levels (**I**).

NE-FSC showed a strong correlation with toxic changes in the pyometra (r = 0.6436, p_S_ = 0.002, **) (Figure 6C) and sepsis groups (r = 0.5884, p_S_ = 0.005, **) (Figure 6A). With the count of bands, NE-FSC showed a strong correlation in the sepsis group (r = 0.6147, p_S_ = 0.007, **) (Figure 6B) and a non-significant correlation in the pyometra group (r = 0.3180, p_S_ = 0.198, ns) (Figure 6D). NE-FSC did not correlate significantly with CRP in the pyometra group (r = 0.4430, p_S_ = 0.050, ns) (Figure 6E). Beyond these findings, no other parameters demonstrated a moderate or strong correlation with NE-FSC in the sepsis and pyometra groups. There was no moderate or strong correlation between NE-FSC and any other parameter in the SRMA or idiopathic epilepsy groups. See also Figure 7.

## 4. Discussion

This study aimed to evaluate the diagnostic potential of the novel neutrophilic granulocyte parameters—NE-SFL, NE-SSC, and NE-FSC—measured by the Sysmex XN-1000V hematology analyzer, in distinguishing between inflammatory and non-inflammatory conditions in dogs. Our results demonstrate that these parameters, especially NE-SFL and NE-SSC, have promising value as indicators of inflammation in dogs. The establishment of a control group of healthy dogs for NE-SFL, NE-SSC, and NE-FSC represents a crucial first step toward the introduction of these parameters into clinical veterinary diagnostics. The defined value ranges provide a baseline for comparison and serve as an initial orientation to guide future studies in assessing their clinical significance. Notably, control samples did not require manual gating, supporting the robustness of these measurements in healthy dogs.

In the clinical part of the study, dogs diagnosed with inflammatory diseases, such as sepsis and pyometra, showed significantly higher levels of NE-SFL and NE-SSC compared to healthy controls. Furthermore, these parameters were also significantly increased relative to non-inflammatory cases (idiopathic epilepsy). NE-FSC was only significantly increased in the sepsis group, suggesting its utility might not be very specific to certain types or severities of inflammation.

Among the three parameters, NE-SFL showed the most consistent and significant differentiation across the tested disease groups. It was elevated in all inflammatory conditions and showed clear separation from both healthy controls and dogs with idiopathic epilepsy. This supports previous findings in human medicine, where NE-SFL, as part of the ICIS, has been shown to significantly discriminate between patients with infection and patients without infection [2,3]. The fluorescence signal reflects increased nucleic acid content due to enhanced cellular activity and correlates well with the presence of toxic changes in neutrophils, which are commonly seen during inflammation [34], and increased numbers of bands. It also aligns with previous findings, which showed that NE-SFL was significantly increased in dogs with systemic inflammation and positively correlated with CRP concentrations [12]. In contrast to the study by O’Toole et al. the present study showed that Ne-SFL and CRP concentrations did not correlate. This might be due to the low number of animals within each disease group, whereas O’Toole et al. included various diseases in one large group. This study further distinguishes itself by incorporating manual gating of samples, focusing on smaller yet clearly defined disease groups, and including a healthy control group for comparison.

NE-SSC, reflecting the granularity and internal complexity of neutrophils, was also significantly elevated in inflammatory conditions. This is supported by the fact that NE-SSC has shown a strong correlation with toxic changes in the pyometra and sepsis groups, suggesting that structural and morphological changes in neutrophils accompany activation and toxic transformation.

NE-FSC, which correlates with neutrophil size, was only elevated in the sepsis group but not in pyometra, SRMA or idiopathic epilepsy cases. This could imply that NE-FSC is more reflective of neutrophil swelling or cellular enlargement, a change possibly more characteristic of bacterial infections. This would be consistent with the fact that NE-FSC correlated with toxic changes and the count of bands in the sepsis and pyometra groups. Its lack of elevation in SRMA cases suggests that NE-FSC may not be as sensitive in detecting sterile, immune-mediated inflammation. Dogs with idiopathic epilepsy, representing the non-inflammatory group, exhibited NE-SFL, NE-SSC, and NE-FSC values within the value ranges of the control group. This confirms that the novel neutrophil parameters are not elevated in this non-inflammatory disease, supporting their specificity for inflammatory processes.

The necessity for manual gating in cases of pyometra and sepsis due to abnormal scattergram flags highlights a practical challenge. In severe inflammatory states, increased cytoplasmic toxicity and the presence of bands may disrupt automated classification, requiring manual correction for accurate analysis. This requirement may limit practical application of these parameters in emergency settings since trained staff with the right expertise would be needed to perform the manual gating. Further refinement of automated analysis algorithms or the careful application of the software’s manual gating options are crucial to improve the accuracy and efficiency of these measurements and, therefore, reduce this issue in the future.

The results also align with the current trend in both human and veterinary medicine toward rapid diagnostics that can reduce dependence on time-consuming, costly tests like CRP and PCT. Unlike CRP or PCT, these novel parameters are obtained from the routinely performed WBC analysis using the Sysmex XN-1000V analyzer, thereby eliminating the need for additional blood sampling and providing a practical advantage in routine diagnostics, which represents a step forward in veterinary hematological diagnostics.

A key limitation of this study is the relatively small sample size for each disease category and the healthy control group, which may affect the statistical power and generalizability of the results. Future studies with larger cohorts and a broader range of inflammatory conditions are needed to further validate the diagnostic performance and to establish reference values for these novel parameters. Additionally, longitudinal studies assessing changes in NE-SFL, NE-SSC, and NE-FSC over time in response to treatment could provide insights into their potential role in monitoring disease progression and therapeutic efficiency.

Another potential area of research is the comparison of these novel parameters with existing inflammatory markers in a prospective clinical setting. Evaluating their sensitivity and specificity in combination with traditional diagnostics could help establish optimal diagnostic algorithms for inflammatory diseases in veterinary medicine. Furthermore, investigating their utility in distinguishing bacterial from non-bacterial inflammations could contribute to more targeted antibiotic usage, addressing concerns related to antimicrobial resistance. Future research should also focus on evaluating these novel parameters in juvenile dogs (<1 year) and in Greyhounds.

Additionally, in the future, Delta-H_e_ should also be the target of a similar study. Since it is part of the ICIS in human diagnostics, there might be some potential use in veterinary medicine too. Delta-H_e_ is the difference between reticulocyte (Ret-H_e_) and erythrocyte hemoglobin content (RBC-H_e_) [35]. In human medicine, a negative value for Delta-H_e_ indicates an impaired hemoglobinization of newly formed reticulocytes compared with the hemoglobin content of mature erythrocytes. This suggests that an insufficient iron supply for hemoglobin synthesis could be due to a high-grade inflammation [36]. In veterinary medicine, Delta-H_e_ is especially investigated for its role in differentiating between chronic hemorrhagic and chronic inflammatory anemia, confirming its value as an inflammatory marker similar to humans [37].

## 5. Conclusions

In conclusion, NE-SFL and NE-SSC, and to a lesser extent NE-FSC, show potential as early, accessible, and cost-effective markers of inflammation in dogs. Their integration into routine hematological assessments may enhance early diagnosis and management of inflammatory diseases, ultimately improving patient outcomes; however, the need for manual gating may reduce the speed and practicality of using these parameters in a clinical setting. Continued research and validation in larger populations and across broader disease spectra will be essential to fully realize their clinical potential and establish standardized guidelines for their use in veterinary medicine.

## Figures and Tables

**Table 1:** Minimum, maximum, and median of NE-SFL, NE-SSC, and NE-FSC in the control group (n = 21).

	Minimum	Maximum	Median
**Ne-SFL [ch] ^1^**	38.2	42.7	40.7
**Ne-SSC [ch] ^1^**	92.6	106.6	96.5
**Ne-FSC [ch] ^1^**	38.7	53.4	41.8

^1^ ch = channel value (technical value, describing position in the axis).

**Table 2:** Overview of median and interquartile range (IQR) in different disease categories and in the control group with corresponding reference intervals.

	Reference Intervals	Healthy (N = 21)		Sepsis (N = 21)		Pyometra (N = 20)		SRMA (N = 23)		Idiopathic Epilepsy (N = 20)	
		Median	IQR	Median	IQR	Median	IQR	Median	IQR	Median	IQR
**CRP [mg/L]**	≤10.7 (Randox) ≤10.2 (Gentian)	5	0	164.7	139.5	280.5	181.15	173.5	120.1	5	0
**WBC [×10^3^/L]**	4.7–11.3 (5%, 95% quantile)	8.29	1.12	14.91	18.67	21.78	14.82	21.54	8.22	7.98	3.4
**Bands [×10^3^/µL]**	0–0.084 (min.–max.)	-	-	1.55 N = 18 ^2^	2.30 N = 18 ^2^	1.78 N = 18 ^2^	3.07 N = 18 ^2^	0.48 N = 7 ^2^	0.32 N = 7 ^2^	-	-
**NE-SFL [ch] ^1^**	38.2–42.7 (min.–max.)	40.7	2.8	68.1	38.6	76.45	26.05	45.8	3.65	39.5	2.28
**NE-SSC [ch] ^1^**	92.6–106.6 (min.–max.)	96.5	4.8	109.7	10.2	106.6	12.3	100.2	7.15	95.9	7.12
**NE-FSC [ch] ^1^**	38.7–53.4 (min.–max.)	41.8	2	44.4	7.3	45.1	6.2	41.3	1.95	41.35	1.85

^1^ ch = channel value (technical value, describing position in the axis) ^2^ N = number of dogs in which bands were present.

**Table 3:** Overview of toxic changes in different disease categories and in the control group.

	Healthy (N = 21)	Sepsis (N = 21)	Pyometra (N = 20)	SRMA (N = 23)	Idiopathic Epilepsy (N = 20)
**Toxic changes:** **-Quantity [%]**	-	5–10% (N = 4) ^1^ 11–30% (N = 3) ^1^ >30% (N = 5) ^1^	5–10% (N = 4) ^1^ 11–30% (N = 4) ^1^ >30% (N = 5) ^1^	5–10% (N = 1) ^1^ 11–30% (N = 0) ^1^ >30% (N = 0) ^1^	-
**-Degree [1+–3+]**	-	1+ (N = 12) ^1^	1+ (N = 8) ^1^ 2+ (N = 5) ^1^	1+ (N = 1) ^1^	-

^1^ N = number of dogs in which toxic changes were present.

## Data Availability

The dataset is available upon reasonable request from the authors.

## Abbreviations

The following abbreviations are used in this manuscript:

ch	Channel value
CRP	C-reactive protein
CT	Computed tomography
Delta-He	Ret-He minus RBC-He
ICIS	Intensive Care Infection Score
IQR	Interquartile range
K3EDTA	Tripotassium ethylenediaminetetraacetic acid
MRI	Magnetic resonance imaging
N	Number
NE-FSC	Neutrophil forward scattered light
NE-SFL	Neutrophil side fluorescent light
NE-SSC	Neutrophil side-scattered light
ns	Not statistically significant
PCT	Procalcitonin
pDunn	p-value of Dunn’s test
pKW	p-value of Kruskal–Wallis test
pS	p-value of Spearman’s correlation
RBC-He	Mature red blood cell hemoglobin equivalent
Ret-He	Reticulocyte hemoglobin equivalent
SIRS	Systemic inflammatory response syndrome
SRMA	Steroid-responsive meningitis-arteritis
WBC	White blood cell
WDF	White blood cell differential
